# Resting state EEG abnormalities in autism spectrum disorders

**DOI:** 10.1186/1866-1955-5-24

**Published:** 2013-09-16

**Authors:** Jun Wang, Jamie Barstein, Lauren E Ethridge, Matthew W Mosconi, Yukari Takarae, John A Sweeney

**Affiliations:** 1 Department of Psychiatry, University of Texas Southwestern, Dallas, TX, USA; 2 Department of Pediatrics, University of Texas Southwestern, Dallas, TX, USA; 3 Center for Autism Spectrum Disorders, Bond University, Gold Coast, Australia

**Keywords:** Autism, Resting-state, EEG, Electroencephalography

## Abstract

Autism spectrum disorders (ASD) are a group of complex and heterogeneous developmental disorders involving multiple neural system dysfunctions. In an effort to understand neurophysiological substrates, identify etiopathophysiologically distinct subgroups of patients, and track outcomes of novel treatments with translational biomarkers, EEG (electroencephalography) studies offer a promising research strategy in ASD. Resting-state EEG studies of ASD suggest a U-shaped profile of electrophysiological power alterations, with excessive power in low-frequency and high-frequency bands, abnormal functional connectivity, and enhanced power in the left hemisphere of the brain. In this review, we provide a summary of recent findings, discuss limitations in available research that may contribute to inconsistencies in the literature, and offer suggestions for future research in this area for advancing the understanding of ASD.

## Review

### Introduction

Autism spectrum disorders (ASD) are characterized by social and communication impairments, and by restricted and stereotyped behaviors [1]. ASD affect approximately 1 in 88 children and 1 in 54 males [2]. These disorders are highly heritable, with estimates ranging from 70 to 90% [3], and are known to have a high recurrence rate in siblings (10 to 20% [4]), yet, progress in identifying pathophysiological and etiological mechanisms has been limited.

It is likely that there are many causes of ASD. Several single-gene disorders (for example, Fragile X, tuberous sclerosis) and rare copy number variants (CNVs) (for example, 16p11 deletions, 15q13 duplications) appear to be strongly associated with ASD, but genetic syndromes, mutations, and single-gene etiologies account for only 10 to 20% of ASD cases, and many individuals with these genetic syndromes do not have ASD [5]. The majority of affected individuals appear to have more complex underlying genetic and epigenetic abnormalities, involving highly penetrant yet undiscovered rare mutations, or combinations of less penetrant and more common variants. The highly variable clinical presentation of ASD reflects this heterogeneity. Affected individuals vary greatly in the course of the disorder (around a quarter show significant developmental regression), associated medical conditions, behavioral challenges (for example, sensory issues, hyperactivity), and degree of intellectual impairment [6,7].

Many studies have attempted to characterize the neural system abnormalities associated with ASD [8,9]. Post-mortem studies have most consistently noted abnormalities in the limbic system and cerebellum [10]. Neuroimaging studies have identified abnormalities in brain and head size, and in cerebellum and limbic structures [11-13], with some individuals showing a pattern of early brain overgrowth [11,14-16]. Functional MRI (fMRI) studies have reported abnormalities in individuals with ASD when performing various tasks involving language comprehension [17], working memory [18], face recognition [19,20], and eye movements [9]. Compared with typically developing subjects, individuals with ASD usually express a diffuse network pattern with diminished activity in task-related regions and increased activity in task-unrelated regions [9,20,21]. When there is no task involved, individuals with ASD show functional underconnectivity in anterior-posterior connections [22] and reduced connectivity involving the medial prefrontal cortex and the left angular gyrus [23]. Moreover, a lack of deactivation in task-related regions during rest has also been reported in individuals with ASD [24]. Although post-mortem and structural MRI studies of ASD have provided promising insights, abnormalities often fail to provide a direct link to the clinical symptomatology of the disorder, with few exceptions [13,25], a challenge for which neurophysiological studies offer advantages.

Electroencephalography (EEG), which primarily measures neurophysiological changes related to postsynaptic activity in the neocortex [26], has proven to be a powerful tool for studying complex neuropsychiatric disorders [27-30]. EEG has been the primary measure used to capture and characterize epileptiform and abnormal paroxysmal activity through the detection of focal spikes, which occur with increased frequency in ASD [31,32]. Resting EEG studies have shown that 20% of individuals with ASD show epileptiform discharges at rest, typically without the presence of clinical seizures [33,34]. Higher rates of epileptiform activity have also been reported in sleep studies; for example, Chez, *et al*. [35] reported that 61% of individuals with ASD and no clinical history of seizures displayed epileptiform abnormalities.

The most common way to characterize resting EEG is by breaking down the oscillatory patterns into bands of frequencies that share physiological properties. The typical clinically relevant frequency bands of EEG range from 0.3 to 100 Hz. Within the scope of the current paper, we focus on five frequency bands ranging from 1 to 100 Hz: delta (1 to 3 Hz), theta (4 to 7 Hz), alpha (8 to 12 Hz), beta (13 to 35 Hz), and gamma (>35 Hz). These historically documented frequency bands have attracted rapidly growing interest in clinical and cognitive neuroscience fields, and are believed to govern different cognitive processes [36]. Delta dominates deep sleep, and is thought to underlie the event-related slow waves seen in tasks for detection of attention and salience [37]. Theta is most commonly studied in relation to memory processes [38]. Alpha waves are present in relaxed awake individuals, and are associated with precise timing of sensory and cognitive inhibition [39]. Beta waves are associated with alertness, active task engagement, and motor behavior [40]. Finally, gamma waves, present during working-memory matching [41] and a variety of early sensory responses, are believed to facilitate feature binding in sensory processing [42,43].

Additionally, EEG recordings can be used to assess functional connectivity between different brain regions over time via EEG coherence, and quantitative measurement of the relationship of frequency spectra between two EEG signals [44]. This advantageous feature can further our understanding of the impaired interactions between brain regions of individuals with ASD that have been suggested by functional MRI studies [45-51].

### Advantages of resting-state EEG for studying brain dysfunction in ASD

Resting-state EEG studies are used to monitor brain activity in the absence of overt task performance or sensory stimulation. These measurements can identify abnormalities for which evoked potential studies, the most widely used approach in EEG research with ASD, are not well suited [52]. Indeed, task-dependent changes in brain function are difficult to interpret without fundamental knowledge of functional differences in individuals with ASD at rest. In task-based evoked potential studies, only time-locked neural responses to events of interest are studied; all other spontaneous activity is typically considered background noise [53-55]. Multiple studies have suggested that the brain is a system that operates intrinsically, with intrinsic resting-state integration. External sensory information interacts with, rather than determines, the operation of brain systems [55-57]. For example, many studies have shown that pre-stimulus EEG activities predict the event-related potentials for visual stimuli [58] or motor responses [59].

There are also several practical advantages of using EEG to study brain function in developmental disorders such as ASD. Compared with MRI, EEG can be used across a wider range of age groups and developmental abilities to study brain physiology, has a higher relative tolerance for movement, has higher temporal resolution, is more clinically available, and can be used to collect repeated measurements because (compared with positron emission tomography) it is non-invasive. Resting-state approaches do not require subjects to make a response. This element is particularly promising for studying more severely impaired and/or younger patients who may not be able to perform tasks accurately because of cognitive, physical, or developmental challenges. This is crucial for studying the abnormal maturational trajectory in ASD through early childhood. The literature on resting-state EEG in healthy individuals shows increased alpha power and coherence in individuals with ASD [60], as well as reduced power in low-frequency bands (delta, theta) [61] in adults relative to children. These findings reflect maturation of long-range cortico-cortical connections into adulthood.

Quantitative EEG of resting-state data also has promise as an approach for monitoring treatment outcomes. Pineda *et al*. [62] reported that individuals with ASD who received neurofeedback training on controlling neural oscillatory activity in the alpha or mu band displayed decreased mu power and coherence, as well as improved performance on an attention test and decreased scores on the Autism Treatment Evaluation Checklist [63]. Neurofeedback training (aiming at reducing theta activity while increasing beta activity) has also been reported to improve executive test performance in individuals with ASD (including attentional control, cognitive flexibility, and goal-setting) for up to 12 months [64,65].

Despite these unique advantages, relatively few studies have used EEG to study resting-state brain alterations in ASD. In the present article, we review the existing literature on EEG resting-state abnormalities in ASD, discuss potential causes of inconsistencies between studies, and offer suggestions for future studies utilizing resting-state EEG to understand the pathophysiological mechanisms involved in ASD.

### Resting-state EEG findings in ASD

Early resting-state EEG studies of ASD failed to identify consistent patterns of atypical neural activity [66-70]. The recognized prevalence of EEG abnormalities in patients varied greatly between studies, which may be attributable to the lack of standardized diagnostic approaches at the time or to limitations in EEG recording technology (for example, small numbers of electrodes) and analysis (for example, qualitative judgments, different approaches to quantification). We limited our review to EEG studies that used spectral analysis to investigate activity in different power bands and coherence between hemispheres and brain regions (Table 1).

**Table 1:** Power and coherence effects in ASD compared with typically developing individuals

**Frequency band**	**Brain region(s)**	**Effect**	**Ref(s)**
		**Absolute power**	
Delta	Frontal	Enhanced	[74,75]
	Frontal	*Reduced*	[77,80]
	Central/parietal	Enhanced	[73]
	Temporal	*Reduced*	[80]
Theta	Frontal/prefrontal	Enhanced	[75,78]
	Frontal	*Reduced*	[80]
	Temporal	*Reduced*	[80]
	Parietal	*Reduced*	[80]
Alpha	All regions	No effect	[77,105]
	Frontal/prefrontal	*Reduced*	[80]
	Frontal	Enhanced	[106]
	Parietal	Enhanced	[106]
	Central	Enhanced	[106]
	Temporal	*Reduced*	[80]
Beta	All regions	No effect	[80]
	All regions	*Reduced*	[77]
Gamma	Midline/central and parietal	Enhanced	[79]
		**Relative power**	
Delta	All regions	Enhanced	[72]
	Frontal	*Reduced*	[77]
	Central/parietal	Enhanced	[73]
Theta	Frontal/prefrontal	Enhanced	[76]
	Right posterior	Enhanced	[77]
Alpha	All regions	*Reduced*	[72,73]
	All regions	No effect	[77]
	Frontal/prefrontal	*Reduced*	[76]
	Occipital/parietal	*Reduced*	[76]
Beta	Occipital/parietal	Enhanced	[76]
		**Coherence**	
Delta	Short/long intrahemispheric	*Reduced*	[77]
	Lateral-frontal intrahemispheric	Enhanced	[118]
	Middle frontal	*Reduced*	[118]
	Occipital	*Reduced*	[118]
	Frontal	*Reduced interhemispheric*	[77]
	Temporal	*Reduced interhemispheric*	[77]
	Central/parietal/occipital	*Reduced interhemispheric*	[77]
Theta	Short/long intrahemispheric	*Reduced*	[77,119]
	Short/long intrahemispheric	Enhanced	[76]
	Frontal	*Reduced interhemispheric*	[77]
	Temporal	*Reduced interhemispheric*	[77]
	Central/parietal/occipital	*Reduced interhemispheric*	[77]
Alpha	Frontal	*Reduced*	[76]
	Between frontal and all other regions	*Reduced*	[76]
	Temporal	*Reduced interhemispheric*	[77]
	Short/long intrahemispheric	*Reduced*	[119]
Beta	Central/parietal/occipital	*Reduced interhemispheric*	[77] ,
	Frontal-temporal	*Reduced*	[119]
	Short/long intrahemispheric	*Reduced*	[119]
		**Hemispheric asymmetry**	
Delta	Frontal	Reduced power in left hemisphere	[80]
	Frontal	Enhanced power in left compared with right hemisphere	[74]
	Temporal	Reduced power in left hemisphere	[80]
	Temporal	Enhanced power in left compared with right hemisphere	[74]
	Parietal	Enhanced power in left compared with right hemisphere	[74]
	Posterior-temporal	Enhanced power in left compared with right hemisphere	[72]
	Central	Enhanced power in left compared with right hemisphere	[72]
	Occipital	Enhanced power in left compared with right hemisphere	[72]
	Occipital	No difference between left and right hemispheres	[105]
Theta	Frontal	Reduced power in left hemisphere	[80]
	Frontal	Enhanced power in left compared with right hemisphere	[74,78]
	Temporal	Reduced power in left hemisphere	[80
	Temporal	Enhanced power in left compared with right hemisphere	[74]
	Parietal	Enhanced power in left compared with right hemisphere	[74]
	Right posterior	Enhanced power in right hemisphere	[77]
	Posterior-temporal	Enhanced power in left compared with right hemisphere	[72]
	Central	Enhanced power in left compared with right hemisphere	[72,74]
	Occipital	Enhanced power in left compared with right hemisphere	[72, [74]
	Occipital	No difference between left and right hemispheres	[105]
Alpha	Frontal	Reduced power in left hemisphere	[80]
	Mid-frontal	Enhanced power in left compared with right hemisphere	[106,107]
	Temporal	Reduced power in left hemisphere	[80]
	Temporal	Enhanced power in left compared with right hemisphere	[74]
	Parietal	Enhanced power in left compared with right hemisphere	[74]
	Posterior-temporal	Enhanced power in left compared with right hemisphere	[72]
	Central	Enhanced power in left compared with right hemisphere	[72]
	Occipital	Enhanced power in left compared with right hemisphere	[72]
	Occipital	No difference between left and right hemispheres	[105]
Beta	Posterior-temporal	Enhanced power in left compared with right hemisphere	[72]
	Central	Enhanced power in left compared with right hemisphere	[72]
	Occipital	Enhanced power in left compared with right hemisphere	[72]
	Occipital	No difference between left and right hemispheres	[105]
Mu	Central	No difference between left and right hemispheres	[74]

### Abnormal power

EEG power can be measured as either relative power or absolute power. Relative power is the amount of EEG activity in an individual frequency band divided by the amount of activity in all frequency bands. Absolute power is the amount of EEG activity in one band independent of activity in other bands. Relative power thus reflects the relationship between frequency bands, but does not yield an indication of the degree to which abnormal electrophysiological activity is present in a specific frequency band. Studies of ASD vary widely in the extent to which they present absolute power findings (Table 1), so interpretation of atypical inter-relationships between different frequency bands (relative power) can be advantageous for comparing frequency bands, but also confounds measurement of activity in the target band within any alterations that may occur in other frequency bands. Absolute power is in many ways preferable for developing an understanding of electrophysiological alterations in ASD, and for the interpretation of differences in relative power in this population [71].

**Figure 1:**
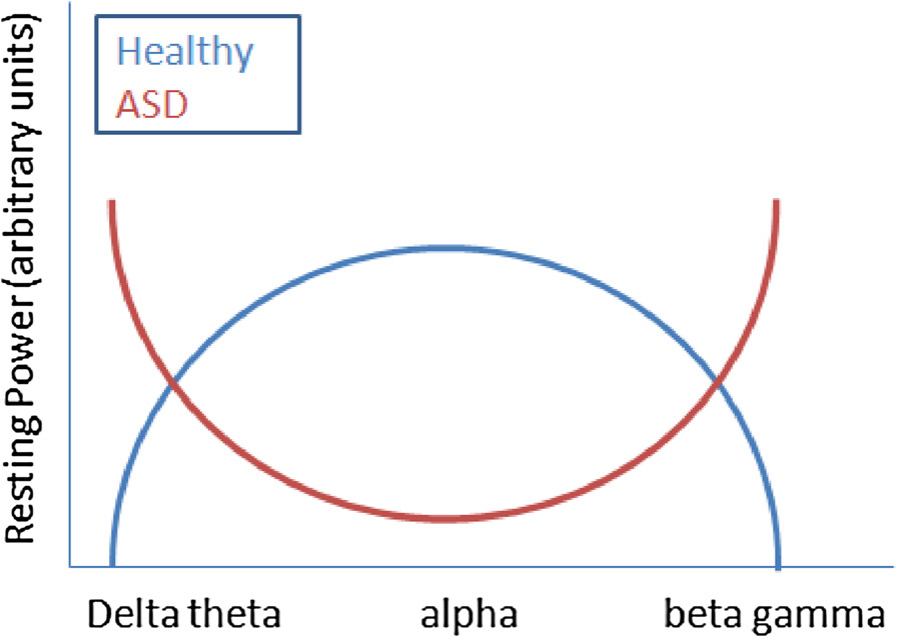
Illustration of the U-shaped profile of abnormal power pattern in autism spectrum disorders (ASD).

In addition to differences between studies in approaches for characterizing EEG activity, studies of ASD are also confounded by the extreme behavioral and putative neurophysiological heterogeneity that characterizes this disorder. Studies vary widely in the demographic characteristics of their samples, and factors such as the age of subjects studied, and whether or not subjects with intellectual disability (ID) were included, may significantly affect study findings. Despite these important concerns, a relatively consistent and unique profile of electrophysiological abnormalities has emerged from resting-state EEG studies, which appears to be present across diverse patient populations. Specifically, excessive power at low-frequency (delta, theta) and high-frequency (beta, gamma) bands, but reduced power in the middle-range frequency band (alpha) (Figure 1) has been found at all stages of development and in children with and children without comorbid ID [72-75]. The excess in delta power has been found in both relative [72,73] and absolute [73-75] powers, and in multiple brain regions, including the dorsal midline, parietal, right temporal [73] and frontal cortical [74,75] areas, suggesting a widely distributed pattern of abnormality [72]. Similarly, enhanced low frequency relative [76,77] and absolute [75,78] theta (4 to 7 Hz) activity has been seen in both adults [76,78] and children [75,77] with ASD, primarily in the frontal and right posterior cortex. Enhanced power has also been reported in high-frequency relative beta (13 to 35 Hz) and absolute gamma (>35 Hz) bands in both adults [76] and children [77,79] with ASD. Within the higher-frequency bands, the most significant alterations have been found in gamma power over occipital, parietal [76] and midline [77,79] regions.

In contrast to the excess power displayed in low-frequency (delta, theta) and high-frequency (beta, gamma) bands, individuals with ASD show reduced relative [72,73,76] and absolute [80] power in middle-range (alpha) frequencies across many brain regions [72,73], including the frontal, [76,80], occipital, parietal [76], and temporal [80] cortex. This pattern indicates a U-shaped profile of electrophysiological power alterations in ASD in which the extremities of the power spectrum are abnormally increased, while power in the middle frequencies is reduced. Available evidence for this model is mostly supportive, but more hypothesis-driven work is needed to confirm and validate it.

We speculate that the etiology for this U-shaped profile may be attributed in part to abnormal functioning of gamma-aminobutryic acid (GABA)ergic tone in inhibitory circuitry, which influences the functional and developmental plasticity of the brain and is thought to modulate power in high-frequency and low-frequency bands while increasing the power of middle-range frequencies (alpha band) [81]. Activity in the gamma band that is visible in EEG seizure recordings has been linked to impairment of dendritic GABAergic inhibition [82]. However, increasing GABA concentration by administrating the GABA antagonist vigibatrin has been shown to increase resting delta power in both rats [83] and humans [84], suggesting that a simple decrease in GABA does not fully explain the U-shaped spectral profile in ASD. For example, thalamocortical delta oscillations are produced by an interaction between GABAergic interneurons and N-methyl-D-aspartate receptors on glutamatergic neurons, which are in turn modulated by dopaminergic neurons in the thalamus [85]. ASD power abnormalities may result from a complex pattern of neurochemical alterations that affect the physiology of inhibitory GABAergic inter-neurons and their modulation of excitatory activity in pyramidal cells.

There is evidence that GABAergic interneuron development and connectivity is disrupted in the prefrontal and temporal cortices in ASD [86], and that this disruption is relevant to excitatory/inhibitory balance [87]. The GABA agonist lorazepam has been shown to increase long-range cortical functional connectivity in the alpha and low beta ranges [88], suggesting an association between GABA tone and large scale cortico-cortical connectivity. The middle alpha frequencies have commonly been associated with an ‘idling’ state, or more recently with active inhibition [39,89], a state that has been associated with GABAergic circuitry [90]. GABAergic abnormalities can also have early developmental consequences, as GABA acts as an excitatory trophic factor prenatally, guiding growth and connectivity of dendrites [91]. Abnormal embryonic GABA concentrations could lead to development of abnormal excitatory/inhibitory circuitry, causing long-term alterations in the entire oscillatory activity at multiple frequencies. GABA abnormalities could bias neural networks away from the state of active inhibition (alpha) and towards greater excitation (higher frequencies). Intermittent theta-burst stimulation has been shown to increase cortical inhibition in rat neocortex by reducing parvalbumin expression in fast-spiking interneurons [92]. Stimulation in both the delta and theta frequency bands also increases expression of GABA precursors in inhibitory cortical systems [93]. In such cases, increased low-frequency activity could be a compensatory mechanism in ASD to halt the proliferation of high-frequency excitatory activation produced by GABAergic dysfunction. This hypothesis is also consistent with several studies that have documented GABAergic abnormalities in individuals with ASD [94]. Fatemi *et al*. [95,96] showed reductions in GABA receptor density in cerebellum and Brodmann’s areas 9 and 40 [95,96]. At the genetic level, it has been suggested that an interaction between GABA receptor subunit genes (*GABRA4* and *GABRB1*) could be directly involved in the etiology of ASD by contributing to increased neuronal excitability, particularly during development, when GABRA4 mRNA levels in brain tissues are at their highest [94]. Increased excitation/inhibition ratios reflecting glutamatergic/GABAergic balance [97], induced by endogenously suppressed GABAergic inhibition [98-101], has been shown in some individuals with ASD. Alpha band power is thought to play an important role in top-down control of sensorimotor responses, including successful voluntary inhibition of contextually inappropriate responses [39]. Many children with ASD show increased levels of inattention and impulsivity [102,103], which may be linked to increased rates of inhibitory control errors in affected individuals [104]. Although associations between alpha power and inhibitory control deficits in ASD have not been directly examined, the potential role of this neurophysiological mechanism in this domain of behavioral impairment merits investigation.

There arre inconsistencies between studies regarding the U-shaped pattern of power in ASD. Reduced frontal low-frequency delta has been reported in children with ASD without ID [77] and reduced delta has been seen in the frontal and temporal regions in children with high-functioning and low-functioning ASD [80]. A few studies reported no differences in alpha frequency bands in children with ASD [77,105] and enhanced alpha power over frontal/parietal/midline regions in high-functioning individuals with ASD [106]. Dawson and colleagues [80] found reduced theta band activity in the frontal, temporal, and parietal regions, with no effect in the beta band. Differences in participant characteristics such as IQ and age might account for the inconsistencies in these findings. Within the delta band, all enhanced delta findings (supporting the U-shaped curve hypothesis) have been most robust in relatively low-functioning children (mean IQ = 37*. *[72]), those with over 20% mental age delay [74], and those with 28% lower intelligence score in ASD [73]. However, reduced delta power was reported in high-functioning children with ASD (mean Full Scale Intelligence Quotient of 93) by Coben *et al*. [77]. Findings of reduced alpha power (again supporting the U-shaped curve hypothesis) have been most consistent in low-functioning children with ASD [72,73,80], but there are examples of this pattern in relatively high-functioning adults with ASD [76]. Studies reporting enhanced or unaffected alpha power were reported for high-functioning children with ASD [77,105,106]. The relation between resting EEG abnormalities and level of intellectual disability, impairments in various behavioral domains, history of regression, and other clinical features of ASD will be an important focus of future research in this area as larger, well-characterized cohorts are studied.

### Abnormal hemispheric asymmetry

In addition to spectral power differences in individuals with ASD, changes have been reported in the hemispheric asymmetry of brain neurophysiology. The majority of the existing resting-state EEG literature reports enhanced power in the left compared with the right hemisphere in individuals with ASD, across all frequency bands [72,74,78,106,107]. This asymmetry is generally much larger than the mild individually variable asymmetries seen in typically developing humans [106].

Cantor and colleagues [72] reported that subjects with ASD had enhanced power in the delta band, in the posterior-temporal, midline, and occipital regions of the left hemisphere. Similarly, Stroganova *et al*. [74] found enhanced delta power in the left hemisphere of individuals with ASD in the frontal, temporal, and parietal regions. In the theta band, left-hemisphere dominance in ASD was seen in frontal [74,78], parietal [74], temporal [72,74], and occipital [72,74] regions. In the alpha band, left-hemisphere dominance in ASD was reported in multiple studies in mid-frontal [106,107], temporal, parietal [72,74], midline [72,106], and occipital [72] regions. Finally, Cantor *et al*. [72] replicated the left-hemisphere dominance pattern in the beta band in posterior-temporal, midline, and occipital regions.

Left-hemisphere asymmetry in ASD is of clinical interest, given the common language abnormalities seen in ASD [108-111]. Increased resting power in the left hemisphere may contribute to left-hemisphere performance deficits by decreasing the signal-to-noise ratio during active tasks, similar to reports of increased background noise and behavioral performance impairment in the literature on schizophrenia [112,113]. Left-hemisphere dysfunction may also be dependent on the task that subjects are performing. When performing tasks of executive functioning (for example, Go/No-go and Stroop tests), high-functioning adults with ASD had significantly increased activation restricted to the left hemisphere [114]. Left-hemisphere dysfunction has also been identified in smooth pursuit eye movements in individuals with ASD [115], as have left-lateralized alterations during an oculomotor serial reaction time task [116].

Nevertheless, as in many neuropsychiatric disorders, evidence of lateralized abnormalities has been inconsistent [74]. Dawson *et al*. [80] reported reduced delta power in the left mid-temporal cortex, and Lazarev *et al*. [105] reported no left/right-hemisphere differences frequency bands in the occipital cortex. However, in the same and subsequent reports, the same authors noted hyperconnectivity within the left hemisphere [117] and reduced power in the right hemisphere [105] in children with ASD during presentation of photic driving stimulation. Dawson and colleagues [80] utilized a relatively short (1 second) window to measure delta power, which may affect reliability of measured low-frequency activity. Lazarev and colleagues [105,117] measured activity only in 14 relatively heterogeneous subjects with ASD, so those studies may have lacked statistical power to detect effects. Photic driving is a robust response, and would potentially be more sensitive to small pathological alterations in studies with small patient cohorts.

### Abnormal coherence

Resting-state EEG studies of ASD have also documented reduced long-range coherence patterns [76,77,118,119]. Weaker coherence between frontal and occipital regions was reported for delta [77,118] and theta [77] bands, whereas Murias and colleagues [76] reported significantly reduced alpha coherence between the frontal cortex and the temporal, parietal, and occipital cortices. Duffy and Als [119] reported weaker left frontal-temporal connectivity within the beta band. This finding is similar to results from multiple fMRI studies that have shown reduced left frontotemporal connectivity during resting state [18,46,120]. These findings parallel those from Horwitz and colleagues [121], who used positron emission tomography to show reduced correlations in glucose metabolism between frontal and other cortical areas in resting adults with ASD. Generalized decreases in frontoparietal and fronto-occipital connectivity have also been reported in ASD during resting fMRI [22,50]. These findings converge to suggest weakened long-range connectivity between the frontal lobe and other cortical regions. The frontal lobe plays a crucial role in higher-order cognitive, language, social, and emotional functioning [122]. Thus, it is not surprising that clear deficits in frontal lobe connectivity have been reported in ASD, as frontal lobe abnormalities have been proposed to play a key role in regulating a wide range of cognitive, sensory, and motor processes [123-125].

Findings on short-range connection patterns in resting EEG studies are less consistent. Both intrahemispheric and interhemispheric local coherences in all brain regions have been reported to be reduced in delta and theta bands [77], while a reduced local coherence over mid-frontal regions has been found in both the delta [118] and alpha band [76]. By contrast, enhanced local coherence has been found over the lateral-frontal region in the delta band [118], and over left frontal and temporal regions in the theta band [76]. Furthermore, these functional frontal deficits have been linked to structural abnormalities in the frontal lobe in ASD [126]. Although several studies have suggested excessive short-range connectivity in ASD, due to increased density of cortical mini-columns [123] and disproportionately increased white matter [127,128], some diffusion tensor imaging (DTI) studies failed to report this pattern [49]. Although short-range coherence studies will require more investigation with newer high-resolution DTI techniques, in parallel with resting-state measures of frontal connectivity using EEG, weaker long-range coherence between frontal and other brain regions found in resting EEG studies suggests that the frontal lobe is less well integrated with other local cortical areas, in ways likely to have considerable neurobehavioral significance [123]. Further examination of this model is necessary to understand how these functional abnormalities relate to clinical phenotypes.

### Crucial considerations

Although previous studies of resting-state EEG in ASD have identified abnormalities in low-frequency and high-frequency band power, connectivity, and lateralization of brain functions, there are multiple crucial methodological and clinical issues that warrant attention in reviewing this literature and in planning future research.

#### Small sample size with narrow range of subject characteristics

Many previous studies were conducted with small sample sizes (often with <20 subjects per group), as well as with subjects displaying a narrow range of demographic/clinical characteristics including age, intellectual ability, history of seizures, and severity of behavioral problems. Developmental variations in power at different frequencies, coherence, and lateralization of function are important considerations when studying a developmental disorder such as ASD, in which behavioral and cognitive presentations are diverse and can change over the age span [129,130]. Additionally, individuals with ASD vary widely in the extent to which their intellectual abilities are affected. IQ and educational levels were not consistent between previous studies of resting-state EEG, and at times, not even between patient and control groups. Other clinical aspects could also affect profiles of neurophysiological alterations in ASD, including comorbid medical and psychiatric conditions.

Many investigators did not conduct correlational analyses to relate abnormal EEG patterns to severity of various clinical aspects of ASD. This limits understanding of the clinical relevance of EEG observations. Of those studies reporting clinical correlations, Orekhova *et al*. [79] found a positive correlation between gamma activity and cognitive delay in ASD. Sutton *et al*. [106] reported that abnormal left frontal asymmetry, defined by greater activation in the left frontal regions, was related to higher levels of social anxiety and social stress, as was abnormal right frontal asymmetry. Stroganova *et al*. [74] showed that increased prefrontal delta power was related to cognitive delay in ASD. Burnett *et al*. [107] reported an association of left frontal EEG asymmetry with parental reports of later onset of ASD symptoms, and increased instances of aggressive outbursts and obsessive compulsive behavior. Finally, Barttfeld *et al*. [118] described a positive correlation between short-distance synchronization and Autism Diagnostic Observation Schedule (ADOS) scores [131], with a negative correlation between long-distance synchronization and ADOS scores, suggesting that there may be a clinically relevant excess of short-range func-tional connectivity coupled with a reduction in long-distance connectivity across the brain in ASD.

In future studies, it will be of great value to utilize large samples with a wide range of subject characteristics to increase the range of brain alterations and to better establish clinicopathological associations.

#### Resting-state conditions

Although the literature reviewed above referred to testing of subjects in the resting state, different studies have used eyes-closed (EC) and eyes-open (EO) conditions. In the EO condition, subjects typically viewed calming stimuli such as bubbles moving across a screen. Distinct EEG patterns in these two conditions were recently reported. Barry *et al*. [132] reported significant amplitude reductions in delta (lateral-frontal), theta (posterior), alpha (posterior), and beta (posterior) frequency bands in EO conditions relative to EC conditions. By contrast, increased frontal beta was found in EC conditions. Furthermore, skin conductance levels were higher in EO conditions, and were negatively correlated with alpha power, indicating a higher level of arousal. Chen *et al*. [133] reported enhanced prefrontal delta and reduced frontal-midline theta in EO states. Finally, reduced low alpha and low beta (13 to 23 Hz) in the posterior region was reported during the EO condition, whereas high beta (24 to 34 Hz) and gamma failed to show any difference between conditions. In evaluating the use of EO versus EC conditions in ASD, a recent study by Mathewson *et al*. [134] reported that adults with ASD did not differ from healthy controls on alpha power levels in the EC condition but displayed less alpha suppression during the EO condition. Although Barry and colleagues later replicated their resting EEG results for healthy adults [132] and children [135], a direct comparison of resting-state conditions has not been done in children with ASD, to our knowledge, so it is unclear whether a similar pattern would be seen in this population.

#### EEG analysis methods: confounds and suggestions

EEG studies need to be concerned about blurring sources of neural activity on EEG scalp recordings, due to the highly conductive nature of the scalp and differences in electrical conductivities between the brain, cerebrospinal fluid, and skull. Inhomogeneities in conductivity between tissues can change patterns of volume conduction (transmission of the electrical signal from the source to the measurement electrode), particularly when source models are calculated based on standard assumptions of tissue thickness and position, rather than the more realistic but not always available individual structural MRI measurements. These blurred recordings make it difficult to identify the source of atypical spectral activity, especially when fewer electrode leads are used in studies. Although the most consistent pattern of findings in the ASD literature is a U-shaped pattern of spectral power relative to healthy controls, the reported topographical locations of frequency band alterations in ASD have been disparate. For instance, in the delta band, significant power difference was presented in frontal [74,75], midline/parietal [73], temporal [80] or even all regions [72]. These widespread differences in topography could be due to widely distributed deficits within each frequency band, or due to data blurring, an issue that could be improved by comparing source densities between groups. In fact, in a source localization study on EEG oscillations [136], focal sources were reported for different frequency bands (for example, the most anterior source for delta and the most posterior source for alpha). However, resting frequency bands often show a distributed source network during simultaneous EEG-fMRI [137,138], so the possibility that multiple sources within each frequency band may be contributing to differential findings between studies needs to be considered.

In addition to its effect on power, data blurring also has a large effect on coherence analysis, especially for short-range coherence. This could contribute to the mixed results for short-range coherence patterns that exist between resting-state EEG studies. Two techniques may help to resolve this blurring problem. One technique includes the use of surface Laplacian transformation to estimate current source density (CSD) based on EEG potentials, from which power and coherence can then be evaluated. CSD transformation computes the second spatial derivative of voltage between nearby electrode sites. This approach can enhance contributions from local electrical activity while attenuating contributions from remote activity (although care should be taken interpreting results if deep sources are of interest, as this technique is necessarily biased toward superficial cortical tissue). A recent resting EEG study with a large sample size utilized this technique to overcome the spatial blurring limitation, and found an overall reduction in short-range connectivity in ASD [119]. The second technique was proposed by Hoechstetter and colleagues [139], and in this technique, surface potentials are first transferred to source space by using multiple discrete equivalent current dipoles or regional sources. Coherence analysis is then calculated between source regions instead of electrodes. Cornew and colleagues [140] applied this technique in a resting-state magnetoencephalography study in high-functioning children with ASD, although they then quantified local oscillatory activity rather than coherence between regions.

The method of calculation of coherence itself is another important issue. As indicated by Murias *et al*. [76], coherence measured in short distances can be biased by power due to the classic coherence calculation frequently used in resting-state EEG studies. This calculation is a product of complex power spectrum decomposition, and it is sensitive to both amplitude and phase relationships between two signals. Strong power modulation at single sources can be detected by multiple nearby electrodes, inflating local connectivity measurements between these electrodes without reflecting the true coherence between separate but adjacent neural sources [141]. This induces the confounding factor of local source strength, limiting the certainty of the real cause of the relationship when concurrent power modulations are detected (although phase relationship usually has a larger contribution than amplitude [60]). To overcome this limitation, phase synchrony analysis methods represent an approach to assess the phase relationship independently. Lachaux and colleagues [142] proposed calculating a ‘phase-locking value’ (PLV) to measure phase synchrony. In their method, two EEG signals were first narrow band-passed (target frequency ± 2 Hz), then convolved with the Gabor wavelet function. Finally, the phase outputs from wavelet decomposition between two signals were compared. PLV is a metric bound between 0 and 1, with 1 indicating that phase difference varies little between trials (ERP) or segments (resting EEG), and 0 indicating a complete lack of phase synchrony. In addition to measuring phase relationships independently, another important feature is that PLV does not rest on the assumption of stationarity (between trials or segments) as in classic coherence calculation. Stationarity refers to the similarity of spectral properties between measurements, which can be more easily assumed with multiple trials that have identical stimulation periods, as in ERP tasks. In resting EEG, there are no clear breakpoints between segments of continuous data, and segment lengths are often based on the best tradeoff between frequency and temporal resolution, that is, how short each measurement segment can be while still affording accurate coverage of a number of oscillations in the frequency bands of interest. In the case of traditional coherence, non-stationarity of the power in a frequency band across time with no change in phase may present as changes in coherence values. Taking the confound between power and coherence into account is particularly important in studies of ASD, given reports of differences in resting-state spectral power in this population.

EEG recordings acquired in resting-state paradigms include both neural activity and non-neuronal activity such as muscular and cardiac activity, and ocular artifacts (for example, eye movement and eye blink). The conventional visual inspection and epoch rejection used in reviewed resting-state EEG studies may not be sufficient to completely remove these and other artifacts [143,144]. In this scenario, alternative solutions such as independent component analysis (ICA) [53] can serve as a complementary method. ICA is a linear decomposition method that separates a multivariate EEG signal into temporally independent signals available in the raw EEG channel data [145]. Each separate component can be treated like a virtual channel. Within each channel, noise components can be identified through component properties such as topography and spectral characteristics. The application of ICA to remove artifacts has been used with other clinical populations [28,29,146], and can generate relatively artifact-free resting EEG data [119].

Finally, statistical methods such as principal components analysis have proven to be a useful method to distill multidimensional and complex EEG data into more manageable representative components of neural activity patterns [147,148]. This technique has been used to great advantage in coherence modeling in recent investigations of resting EEG in ASD [119].

### Future directions

#### The maturational trajectory of resting-state activity in ASD

Few studies have compared individuals with ASD and healthy individuals, either cross-sectionally between age groups, or longitudinally. A recent longitudinal study of infants at high and low risk for ASD reported changes in developmental trajectory, that is, the slope of the power curve across time, related to risk status from 6 to 24 months of age, particularly in the delta, beta, and gamma frequency ranges [81]. This same group also reported changes in the developmental trajectory of overall EEG complexity (entropy) for high-risk infants as compared with typically developing controls across the same time window [149]. One 3-month longitudinal study in children with ASD indicated that EEG characteristics are relatively stable across short time intervals [150]. Longer-term longitudinal studies are needed to understand whether individuals with ASD show similar trajectories of functional connectivity maturation, or whether these processes are disrupted and/or delayed. Behavioral longitudinal studies of children with ASD indicate an early improvement in language and cognitive skills in some affected individuals (ages 12 to 13 years) followed by considerable and abnormal decrease in the rate of gains through adolescence (ages 19 to 20 years) [151]. These findings raise the possibility that developmental lag or deviance becomes more profound during late childhood and adolescence as the long-range connections to prefrontal cortex are optimized [152]. Reports of resting-state EEG data in younger ASD cohorts are beginning to appear, but studies on a long-term and/or adolescent sample would provide potentially important information about functional connectivity changes accompanying ASD through childhood and adolescence.

#### Early detection and possible biomarkers for ASD

Retrospective studies of infants later diagnosed with ASD have shown that features of ASD are present as early as 12 months. However, many children are not diagnosed until the age of 4 years or later [153,154]. This highlights the need for biomarkers for early detection in order to implement early intervention [155]. It is difficult to reliably identify individuals with ASD within the first year of life based only on behavioral observation, but studies of resting-state EEG suggest that selective alterations may be identifiable as early as 6 months [81,149]. Globally reduced power of delta, theta, alpha, beta and gamma frequency bands have been found in high-risk infants with siblings with ASD compared with low-risk 6-month-old control infants [81]. However, no group difference in hemispheric asymmetry was reported. Elsabbagh *et al*. [156] reported increased gamma band activity in the midline anterior and right temporal cortex of high-risk infants. Currently, there are few studies to determine the utility of these measures in at risk children for identifying individuals with a high likelihood of developing ASD later in life.

#### Resting-state EEG studies in ASD with genetic etiology

The heterogeneous nature of idiopathic ASD can make studies of the underlying etiology difficult. However, a small subset of individuals with ASD (10 to 20%) is believed to have ‘simpler’ genetics, possessing identifiable chromosomal abnormalities or rare mutations found in higher ratios in the ASD population. High proportions of individuals with Fragile X syndrome, Rett syndrome [157], tuberous sclerosis [158], and Phelan-McDermid syndrome [159] have ASD, and each of these disorders has been linked to known abnormalities of individual genes (for example, *FMRI1*, *MECP2*, *TSC1*, *TSC2*). Further, several rare *de novo* mutations, some of which converge on pathways that overlap with those associated with Fragile X and tuberous sclerosis, and are linked to synapse formation and function, have been found to be more common in this population [5]. Using resting-state EEG, it may be possible to connect distinct patterns of altered electrophysiological activity with symptoms found in identified single-gene disorders related to ASD. To date, few studies have utilized ERP in subjects with Fragile X syndrome [160] and primarily epileptiform activity has been examined in resting-state EEG in this population [132,161]; however, there is some evidence for increased resting theta activity in Fragile X [162-164]. The use of EEG to examine Rett syndrome has followed a similar path, with the majority of resting EEG studies dedicated to describing seizure activity (but see studies by Ishizaki [165] and Niedermeyer *et al.*[166] for evidence of prominent resting theta activity in Rett syndrome). Currently, there are no published accounts of resting EEG in Phelan-McDermid syndrome, although changes in excitatory/inhibitory balance in the brains of knockout mouse models suggest measurable electrophysiological changes in this disorder [167]. It would be of particular interest to study resting EEG in these known single-gene conditions, particularly Fragile X, one of the better characterized single-gene disorders, potentially serving as reference for other subpopulations in ASD and shedding light on biological mechanisms shared across the autism spectrum. Whether resting EEG studies can be useful for parsing biological heterogeneity of idiopathic ASD remains another important question to be addressed in future studies.

## Conclusions

In this review, we have addressed resting-state EEG studies in ASD with an emphasis on three aspects: spectral power, coherence, and hemispheric asymmetry. The existing literature suggests a U-shaped pattern of power abnormalities, overall local overconnectivity and long-range underconnectivity, and enhanced power in the left hemisphere of the brain in individuals with ASD. There are important considerations for EEG methodology and clinical assessment that both need consideration for designing the most informative future studies. Recent advances in quantitative EEG analytic methodology and scientific findings from work in this area are encouraging. Future work linking EEG studies of animal models with patient-oriented studies are promising, especially for rare genetic variants for which animal models are most directly relevant. Because of a combination of advantages including its non-invasive nature, high temporal resolution, and relative ease of use across the lifespan, resting-state EEG studies have the potential to make important contributions to the understanding of the pathophysiology of ASD.

## Competing interests

JS serves as a member of advisory boards to Takeda, Lilly, BMS, Roche and Pfizer, and has received support from Janssen that is unrelated to the work presented in this manuscript.

## Authors’ contributions

JW, JB, MW and JS made substantial contributions to design of this study. JW and JB wrote the first draft of the manuscript, and LE, MW, YT and JS revised the manuscript. All authors contributed to writing the manuscript. All authors read and approved the final manuscript.
